# Supplementary material for: Portraying entanglement between molecular qubits with four-dimensional inelastic neutron scattering
Source: Nat Commun. 2017 Feb 20;8:14543. doi: 10.1038/ncomms14543 (PMC5321722; doi:10.1038/ncomms14543)
Supplement: Supplementary Information — Supplementary Figures, Supplementary Notes, Supplementary Tables and Supplementary References [file ncomms14543-s1.pdf]

Supplementary Note 1 - Molecular qubits and Entanglement

Molecular nanomagnets (MNMs) [1] are clusters containing a finite number of paramagnetic ions (typically 3d ions), whose spins are coupled by Heisenberg exchange interactions. Magnetic cores of adjacent molecules are well separated from each other by a surrounding of organic ligands, so that inter-molecular interactions are negligible. Therefore, molecular crystals formed by these kind of metal-organic clusters behave like an ensemble of non-interacting identical molecules and it is possible to detect magnetic properties of a single molecule from bulk measurements. MNMs present several advantages both from a theoretical and experimental point of view: Not only it is possible to describe their magnetic cores in detail with theoretical models, but it is also possible to have an high degree of control of their chemical structure and resulting magnetic properties.

Due to their quantum behavior and properties, MNMs are considered promising candidate to encode qubits, the unit of quantum information for quantum computation algorithms [2]. The Cr_7Ni antiferromagnetic (AF) rings constitute one of the most studied family of molecular qubits [3–6]. The magnetic core is formed by seven Cr ions and one Ni ion arranged at the corners of an octagon. The dominant interaction is the nearest-neighbor antiferromagnetic exchange and leads to an isolated ground doublet behaving as a total spin $S = 1/2$, which can be used to encode a qubit. In addition, excited states can be exploited as additional resources for complex gate operations [7]. These kind of molecules displays coherence times much longer than the single-qubit gate times [6] and they can also be grafted onto surfaces without altering their magnetic properties [8].

Quantum computation algorithms are based on sequences of elementary logical operations on qubits, the so-called quantum gates. In particular, two-qubit gates are based on the control of qubit-qubit interactions. Thanks to targeted ligand design, it has been showed that paramagnetic molecules can implement 2-qubit quantum gates [9]. For instance, the chemically engineered structural asymmetry in Tb_2 molecular clusters renders the two weakly coupled Tb^{3+} qubits magnetically inequivalent, yielding the energy level spectrum needed to realize a CNOT quantum gate [10]. AF rings can be magnetically linked in different ways in supramolecular structures fitting specific quantum information processing schemes. It is possible to control the coupling between molecular spin clusters while preserving the properties of the molecular building blocks [11]. It has been also shown that through supramolecular chemistry that a single simple module, molecular Cr_7Ni rings, can be assembled into structures suitable for implementing either the CNOT or √iSWAP, which are gates that entangle pairs of qubits [12]. Moreover, arrays of molecular nanomagnets can be used as quantum simulators of different model Hamiltonians with translational invariance and short-range interactions [13].

Spin entanglement between individual spins and between two interacting molecular qubits (e.g., AF rings) is a crucial resource for quantum information processes and MNMs are an ideal playground for investigating quantum entanglement in complex spin systems [14]. They represent prototypical examples of correlated, low-dimensional quantum systems and the wide tunability of their physical properties can allow the control of quantum entanglement. In particular, different forms of entanglement can be fine-tuned by chemical processing of well-defined molecular building blocks such as AF rings. For instance, the controlled substitution of single magnetic ions results in the introduction of magnetic defects in otherwise homogeneous molecules, thus affecting correlations between their constituent spins. The growth of supramolecular bridges can instead induce weak exchange couplings between two or more MNMs, to entangle their total spins. It has been theoretically shown [15] how the introduction of magnetic defects by controlled chemical substitutions in the heterometallic Cr_7M rings results in a strong spatial modulation of spin-pair entanglement within each ring, and that in exchange-coupled ring dimers entanglement between local degrees of freedom (individual spins) and collective ones (total ring spins) coexist.

Detection and quantification of entanglement in complex systems represent a challenge from an experimental point of view. For instance, entanglement can be detected by using thermodynamics quantities as entanglement witnesses [16]. Indeed, the violation of the corresponding inequalities allows the detection of entanglement, without requiring the derivation of the system's state, and with a variable amount of knowledge of the system Hamiltonian. In spin systems, like AF rings, magnetic susceptibility can

be used as a witness of the inter-qubit entanglement. In addition, in clusters of antiferromagnetically-coupled spins, exchange energy reflects multipartite entanglement [17]. Supramolecular dimers of linked AF rings are ideal systems to study entanglement between molecular qubits. So far, entanglement in these systems has only been experimentally demonstrated rather indirectly by magnetic susceptibility as entanglement witness [18, 19] and the quantification of the entanglement has been possible only after making specific assumptions on the form of the interaction between the qubits.

Supplementary Note 2 - Anisotropy effects

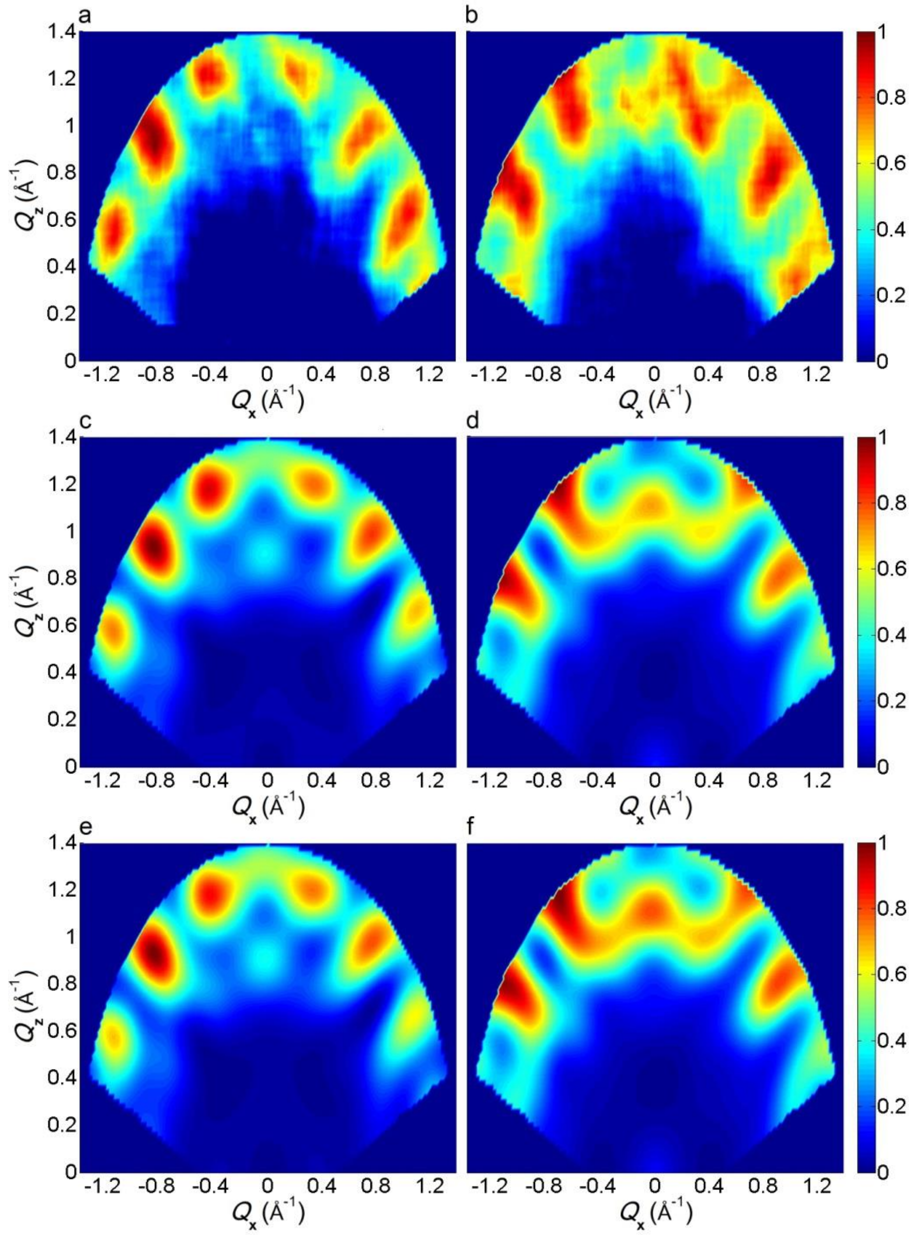

Supplementary Figure 1. Effects of anisotropy on the intensity maps. Constant-energy plots of the neutron scattering intensity of the two observed inelastic excitations. Panels **a** and **b** show the observed dependency of the intensity of the excitations on the horizontal wavevector components Q_x-Q_z, integrated over the full Q_y data range and over energy ranges centred around the observed transition energies: 0.2 meV < E < 0.25 meV for panel **a** and 0.25 meV < E < 0.33 meV for panel **b**. The colour bar reports the transition intensity normalized for the maximum in each panel. Panels **c** and **d** report the corresponding calculations accounting for the presence of differently-oriented dimers in the crystals, obtained with the isotropic model reported in the main text. Panels **e** and **f** show the corresponding calculations obtained by taking into account the axial zero-field splitting anisotropy of each ring.

We have studied the effects of anisotropy on the calculated scattering intensity of the two observed inelastic transitions, by adding the axial zero-field splitting (ZFS) term of the spin Hamiltonian in equation (1) of the main text. ZFS terms have been calculated taking into account the different axial direction (direction perpendicular to the plane) of each single ring in the system with respect to the laboratory reference frame. Results are reported in Supplementary Fig. 1e and 1f, showing that the effects of the anisotropic ZFS terms on the results presented in this work are very small.

Supplementary Note 3 - Ising inter-ring interaction

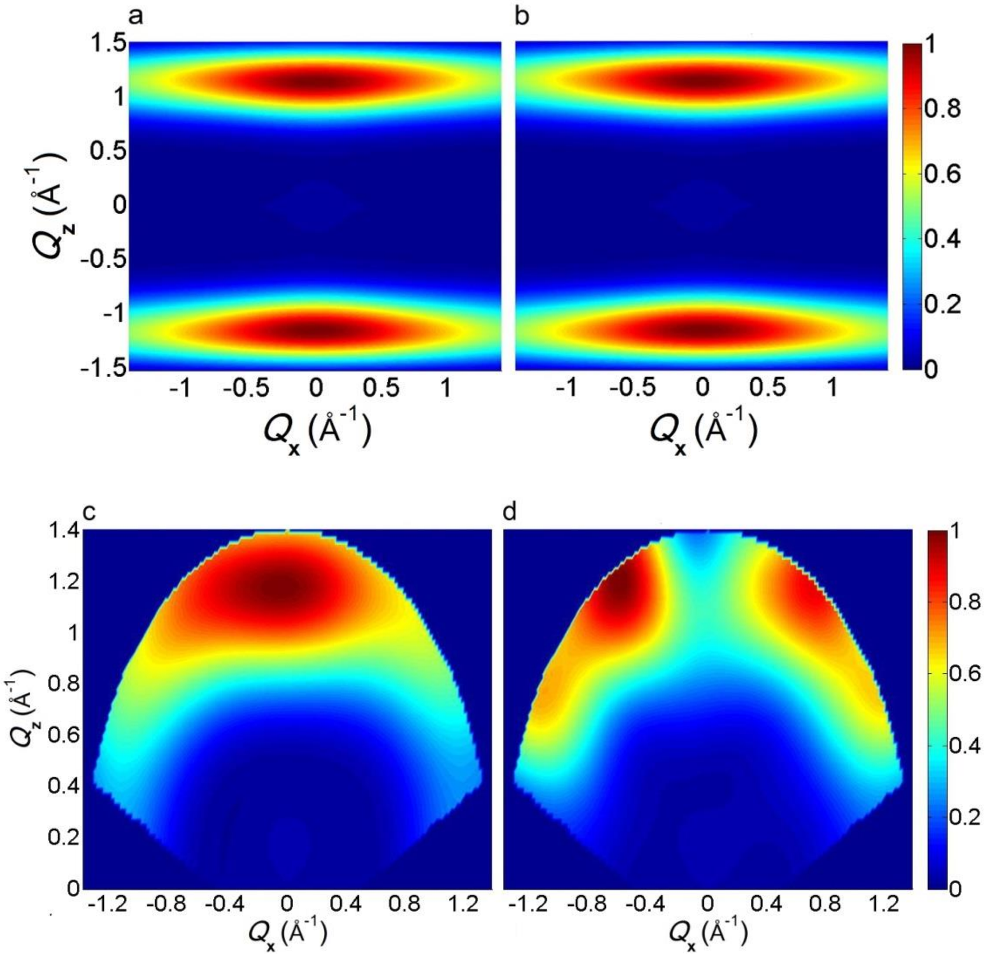

Supplementary Figure 2. Intensity plots with an Ising inter-ring interaction. Calculated neutron scattering intensity in the Q_x-Q_z plane for the two lowest-energy transitions of a single ideal $(Cr_7Ni)_2$ dimer compound with Ising inter-ring interaction (panel **a** $|11\rangle \rightarrow |01\rangle$ and **b** $|11\rangle \rightarrow |10\rangle$). In panel **c** and **d** same calculations are shown for a collection of single crystals of real $(Cr_7Ni)_2$ dimers, performed taking into account the presence of differently oriented dimers in the crystals. In both cases short-Q modulations disappear since the final states of both inelastic transitions are not entangled and there are no inter-ring correlations. The theoretical maps are integrated over the full Q_y experimental range, and are calculated with the temperature and magnetic field used in the experiment. The colour bar reports the transition intensity normalized for the maximum in each panel.

The observation of short-Q modulations in the neutron scattering intensity of $(Cr_7Ni)_2$ due to inter-ring correlations demonstrates that in the excited state the two rings are entangled. Indeed, dynamical correlations between spins belonging to different monomers would be zero if the states of the two monomers were factorized in the probed excited eigenstate (the sizeable field guarantees a factorized

ground state), because the corresponding products of spin matrix elements would be zero in the INS scattering function (see equation (3) in the main text). For instance, an inter-qubit interaction of the Ising type would lead to not-entangled eigenstates for the $(Cr_7Ni)_2$ dimer. Even if the system would yield a similar INS energy spectrum (when the two molecular qubit have slightly different g values), the two excited states would be factorized. Hence, as we show in Supplementary Fig. 2, in this case short-Q modulations in the INS intensity maps disappear. Longer-period modulations in Supplementary Fig. 2 are due to intra-ring correlations in the two non-parallel rings and to the presence of differently oriented dimers in the crystals.

Supplementary Note 4 - Effect of a complex wavefunction

Supplementary Figure 3 reports the comparison between the calculated inelastic neutron scattering intensity maps of the two observed transition, obtained with a real or complex final state |p⟩. The maps have been integrated over and asymmetric Q_y range from -0.2 to 0 Å^{-1}, where it is easier to discriminate between real and complex |p⟩ states, given the present experimental configuration. The comparison with experimental data points to real wavefunctions.

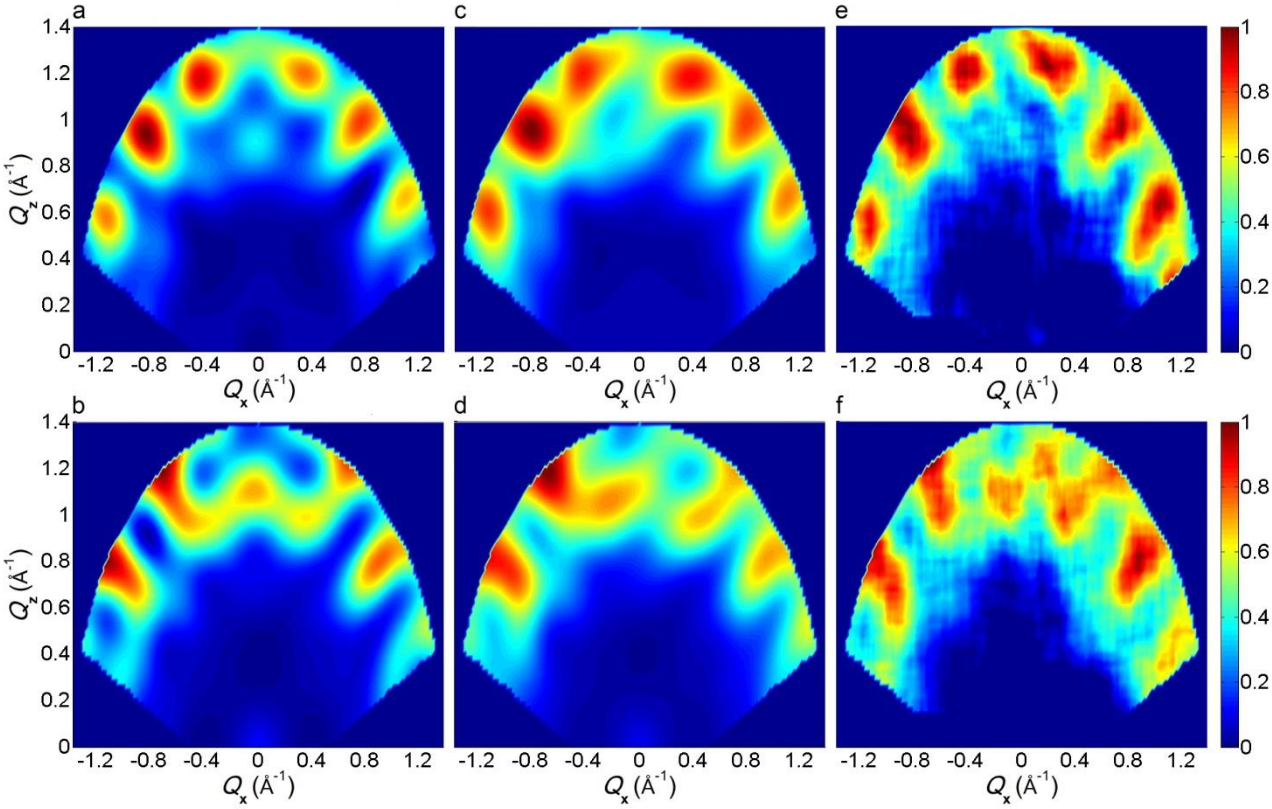

Supplementary Figure 3. Effects of a complex wavefunction on the intensity maps. Calculated (panels **a**, **b**, **c**, and **d**) and experimental (panels **e** and **f**) dependence of the neutron scattering intensity on the wavevector components Q_x-Q_z, integrated over an asymmetric Q_y range. The colour bar reports the transition intensity normalized for the maximum in each panel. We consider different compositions of the excited states |p⟩ with C = 1 with b real and c = $\sqrt{1-b^2}e^{i\varphi}$. In panels **a** and **b** the coefficient c is real (φ = 0), while in panels **c** and **d** the coefficient c is complex (φ = π/3) (d = 0 because of the sizeable applied field). Figures in the first (second) row correspond to the lower-energy (higher-energy) excitation.

Supplementary Note 5 - Crystal structure determinations and refinements

X-ray data were processed and reduced using CrysAlisPro suite of programs. Absorption correction was performed using empirical methods based upon symmetry-equivalent reflections combined with measurements at different azimuthal angles [20-22]. The crystal structures were solved and refined against all F^2 values using the SHELX [23] and Olex2 suite of programs [24]. All the atoms were refined anisotropically. Hydrogen atoms were placed in calculated positions refined using idealized geometries (riding model) and assigned fixed isotropic displacement parameters. Pivalate ligands were disordered and modelled over two different positions. The C-C, C-O distances were restrained using SADI and DFIX commands. The atomic displacement parameters were also restrained using SIMU, EADP and RIGU commands. BUMP command was used to avoid hydrogen short contacts.

Crystal colour	Purple
Crystal size (mm)	0.35 × 0.35 × 0.1
Crystal system	Orthorhombic
Space group, number of molecules per unit cell	$P2_12_12_1$, 4
Unit cell axis *a* (Å)	27.2066(12)
Unit cell axis *b* (Å)	30.7346(17)
Unit cell axis *c* (Å)	31.1172(12)
Unit cell Volume ($Å^3$)	30581(5)
Density ($g.cm^{-3}$)	1.313
X-ray Wavelength (Å)	0.71073
Temperature (K)	150
Absorption coefficient for Molybdenum X-ray radiation μ(Mo-Kα) (mm^{-1})	0.840
2Θ range (°)	6.59 to 61
Reflections collected	91533
Independent Reflections, *n* (R_{int})	63790 (0.0594)
Least square parameters, *p*	2972
Number of restraints, *r*	1535
R1 (*F*) $I > 2.0\sigma(I)$	0.0941
$wR2(F^2)$, all data	0.2590
Goodness of fit $S(F^2)$, all data	1.032

Supplementary Table 1. **Crystal data and refinement parameters.** The table reports the crystal structure information of $(Cr_7Ni)_2$ and the X-ray refinement parameters (see Supplementary Table 2 for parameter definitions).

R1 (F)	$\Sigma(\lvert F_o\rvert - \lvert F_c\rvert)/\Sigma\lvert F_o\rvert$
$wR2(F^2)$	$[\Sigma w(F_o^2 - F_c^2)^2/\Sigma w F_o^4]^{½}$
$S(F^2)$	$[\Sigma w(F_o^2 - F_c^2)^2/(n + r - p)]^{½}$

Supplementary Table 2. Definition of R1, wR2 and goodness of fit. The table reports the definitions of the X-ray refinement parameters of $(Cr_7Ni)_2$, where F_0 are the observed structure factors, F_c are the calculated structure factors and *w* is a weight parameter.

Supplementary References

[1] Gatteschi D., Sessoli R. and Villain J., *Molecular Nanomagnet* (Oxford University Press, New York, 2006).

[2]Troiani, F. *et al.* Molecular engineering of antiferromagnetic rings for quantum computation. *Phys. Rev. Lett.* **94**, 207208 (2005).

[3] Caciuffo, R. et al. Spin dynamics of heterometallic Cr7M wheels (M = Mn, Zn, Ni) probed by inelastic neutron scattering. *Phys. Rev. B* **71**, 174407 (2005).

[4] Troiani, F. et al. Molecular engineering of antiferromagnetic rings for quantum computation. *Phys. Rev. Lett.* **94**, 207208 (2005).

[5] Carretta, S. et al. Quantum oscillations of the total spin in a heterometallic antiferromagnetic ring: Evidence from neutron spectroscopy. *Phys. Rev. Lett.* **98**, 167401 (2007).

[6] Wedge, C. J. et al. Chemical engineering of molecular qubits. *Phys. Rev. Lett.* **108**, 107204 (2012).

[7] Troiani, F., Affronte, M., Carretta, S., Santini, P. & Amoretti, G. Proposal for quantum gates in permanently coupled antiferromagnetic spin rings without need of local fields. *Phys. Rev. Lett.* **94**, 190501 (2005).

[8] Corradini, V. et al. Magnetic anisotropy of Cr_7Ni spin clusters on surfaces. *Adv. Func. Mat*. **22**, 3706- 3713 (2012).

[9] Aromì G., Aguilà D., Gamez, P., Luis F. & Roubeau O. Design of magnetic coordination complexes for quantum computing. *Chem. Soc. Rev.* **41**, 537–546 (2012).

[10] Luis F. et al. Molecular prototypes for spin-based CNOT and SWAP quantum gates. *Phys. Rev. Lett.* **107**, 117203 (2011).

[11] Timco G., *et al.*, Engineering the coupling between molecular spin qubits by coordination chemistry. *Nature Nanotech.* **4**, 173-178 (2009).

[12] Ferrando-Soria J. *et al.*, A modular design of molecular quibits to implement universal quantum gates, *Nature Commun.* **7**, 11377 (2016).

[13] Santini, P., Carretta, S., Troiani, F. & Amoretti, G. Molecular nanomagnets as quantum simulators. *Phys. Rev. Lett.* **107**, 230502 (2011).

[14] Amico, L., Fazio, R., Osterloh, A. & Vedral, V. Entanglement in many-body systems. *Rev. Mod. Phys.* **80**, 517-576 (2008).

[15] Siloi, I. & Troiani, F. Towards the chemical tuning of entanglement in molecular nanomagnets. *Phys. Rev. B* **86**, 224404 (2012).

[16] Horodecki R., Horodecki P., Horodecki M. & Horodecki K., Quantum entanglement. *Rev. Mod. Phys.* **81**, 865-942 (2009).

[17] Siloi I. & Troiani F., Detection of multipartite entanglement in spin rings by use of exchange energy. *Phys. Rev. A* **90**, 042328 (2014).

[18] Candini, A. *et al.* Entanglement in supramolecular spin systems of two weakly coupled antiferromagnetic rings (purple-Cr_7Ni). *Phys. Rev. Lett.* **104**, 037203 (2010).

[19] Troiani F., Carretta S. & Santini P., Detection of entanglement between collective spins. *Phys. Rev. B* **88**, 195421 (2013).

[20] Sheldrick G.M., SADABS, empirical absorption correction program based upon the method of Blessing, University of Göttingen, Göttingen, (1994).

[21] Krause L., Herbst-Irmer R., Sheldrick G.M. & Stalke D., Comparison of silver and molybdenum microfocus X-ray sources for single-crystal structure determination. *J. Appl. Cryst.* **48,** 3-10 (2015).

[22] Blessing R.H., An empirical correction for absorption anisotropy. *Acta Crystallogr.* **A51**, 33-38 (1995).

[23] Sheldrick. G. M., Crystal structure refinement with SHELXL. *Acta Crystallogr*. **C71**, 3-8 (2015).

[24] Dolomanov O.V., Bourhis L.J., Gildea R.J., Howard J.A.K. & Puschmann H., OLEX2: a complete structure solution, refinement and analysis program. *J. Appl. Cryst.* **42**, 339–341 (2009).
